# Clinical and pathological characteristics, diagnosis, and surgical treatment of uterine angioleiomyoma: a retrospective cohort study

**DOI:** 10.3389/fmed.2026.1823201

**Published:** 2026-06-04

**Authors:** Ruirui Li, Wenping Guo, Zibaguli Wubulikasimu, Huaxin Yang, Juan Song, Zhaoli Song, Li Lin

**Affiliations:** 1 Department of Obstetrics and Gynecology, Peking University International Hospital, Beijing, China; 2 Peking University Third Hospital, Beijing, China

**Keywords:** angioleiomyoma, diagnosis, recurrence, surgical treatment, uterus

## Abstract

**Objective:**

Uterine angioleiomyoma (ALM) is a rare benign mesenchymal tumor. Its clinical management poses challenges due to difficulties in preoperative diagnosis, an increased risk of intraoperative bleeding due to its highly vascular nature, and unclear mechanisms and predictive factors for postoperative recurrence. This study aims to describe the clinical and pathological characteristics of uterine ALM, elucidate diagnostic and surgical strategies, and evaluate the safety and efficacy of surgical treatment.

**Methods:**

We conducted a retrospective analysis of 20 patients who were pathologically diagnosed with uterine ALM and underwent surgical treatment at our hospital between August 2015 and August 2023. Data collected included clinical manifestations and signs, imaging findings, surgical procedures, pathological subtypes, and follow-up results. The primary endpoints were symptom relief, intraoperative blood loss, and recurrence.

**Results:**

Only one case was preoperatively suspected as ALM on ultrasound; the remaining cases were diagnosed postoperatively. The median intraoperative blood loss was 15 mL in the 10 patients who underwent hysteroscopy (FIGO type 0–2 submucosal myomas), 300 mL in the 5 patients who underwent open surgery (myoma diameter ≥ 10 cm), and 17 mL in the 5 patients who underwent laparoscopy (myoma diameter < 10 cm). Pathological analysis identified 14 cases of the common type, 3 cases of the degenerative type, and 3 cases of the cellular type. Among the 19 patients who completed follow-up, with a median follow-up duration of 33.5 months, 1 case of the cellular type recurred, and 2 patients achieved spontaneous pregnancy.

**Conclusion:**

The preoperative diagnosis of uterine ALM remains challenging. We recommend a standardized diagnostic workflow: transvaginal ultrasound screening, followed by MRI evaluation, intraoperative assessment, and pathological confirmation. Surgical treatment should be individualized based on lesion location, size, and the patient’s fertility needs. Surgeons should anticipate the potential for significant intraoperative bleeding due to highly vascularized lesions, and complete resection may be important for reducing recurrence based on our observations which requires validation in larger cohorts.

## 1 Introduction

Uterine angioleiomyoma (ALM) is a rare benign mesenchymal tumor, accounting for approximately 0.5%–1.0% of all uterine leiomyomas. Its clinical presentation often overlaps with that of typical leiomyomas, and imaging lacks specific features; consequently, ALM is frequently missed or misdiagnosed preoperatively (1, 2). Histologically, ALM contains abnormal vascular networks, which increase the risk of intraoperative bleeding compared with typical leiomyomas; the mechanisms and risk factors for postoperative recurrence remain unclear (3). Most reports consist of case studies or small series, lacking systematic analyses of clinical characteristics and management (4, 5). We conducted a retrospective analysis of 20 pathologically confirmed cases of uterine ALM from a single center in China, aiming to describe the clinical spectrum of this disease and propose a practical, standardized diagnostic and treatment pathway based on real-world data, including key perioperative risk control measures.

## 2 Materials and methods

### 2.1 Patient selection

We collected clinical data from patients diagnosed with ALM by surgical pathology at Peking University International Hospital between August 2015 and August 2023. Inclusion criteria were: (1) Postoperative pathological confirmation of uterine ALM; (2) Complete clinical, imaging, and follow-up data. Exclusion criteria were: (1) Incomplete imaging data or patients who did not undergo surgical treatment; (2) Concomitant malignant tumors of the reproductive system. Among 25 patients, a total of 20 patients were ultimately included. This study was approved by the hospital’s Institutional Review Board (Approval No.: 2025-KY-0048-02).

Imaging review: All preoperative imaging studies (transvaginal ultrasound and pelvic MRI) were retrospectively reviewed independently by two senior radiologists (with 12 and 15 years of experience in gynecologic imaging, respectively). The reviewers were unaware of the final pathological diagnosis at the time of review. In cases of disagreement (which occurred in 2 of 20 cases, primarily regarding the assessment of tumor margins), consensus was reached through discussion.

Pathological review: All pathological specimens were initially evaluated by the attending pathologist at the time of surgery as part of routine clinical care. Subsequently, all 20 cases underwent central re-review by two senior gynecologic pathologists (with 10 and 18 years of experience, respectively) who were independent of the initial diagnosis and unaware of the clinical and imaging data at the time of re-review. The re-review confirmed the diagnosis of ALM in all cases and included systematic assessment of pathological subtype (common, cellular, or degenerative type), immunohistochemical staining results (SMA, desmin, caldesmon, ER, PR, Ki-67, HMB45), and the presence of mitotic figures. No cases were reclassified following central review.

### 2.2 Patient grouping and surgical selection criteria

ALMs were classified according to the International Federation of Gynecology and Obstetrics (FIGO) classification system (6). Surgical approaches were categorized into three types based on the location and size of the myoma, as well as the patient’s reproductive preferences (7–9).

Hysteroscopic surgery: Indicated for Type 0 submucosal fibroids (including all types of uterine or cervical submucosal fibroids prolapsing into the vagina); Type 1 and Type 2 submucosal fibroids with a diameter ≤ 4–5 cm (8).

Laparoscopic surgery: Indicated for intramural, subserosal, or broad ligament myoma of types 3–8, with a diameter < 10 cm, no severe pelvic or abdominal adhesions, and no malignant features.

Open surgery: Indicated for myomas with a diameter ≥ 10 cm, suspected malignant features, multiple giant myomas, or cases where severe pelvic or abdominal adhesions are suspected.

All surgeries were performed by senior physicians.

For 10 patients who underwent hysteroscopic resection of submucosal myoma the following standardized surgical protocol was adopted. All procedures were performed under general anesthesia with the patient in the lithotomy position. On the evening prior to surgery, for patients without active vaginal bleeding, 200 μg of misoprostol was administered via the posterior fornix to promote cervical ripening. A bipolar hysteroscopic resectoscope (Olympus, Japan; outer sheath diameter 9 mm) was used, with 0.9% saline as the distension medium. The intrauterine pressure was set at 80–100 mmHg, and the flow rate at 400 ml/min. Real-time transabdominal ultrasound guidance was employed throughout the procedure. The lesion was resected using the resectoscope’s ring electrode, and larger fragments were removed using oval forceps to shorten the operative time. Oxytocin was administered intraoperatively to promote protrusion of the myoma into the uterine cavity. Fluid absorption (the difference between inflow and outflow of perfusion fluid) was closely monitored and consistently maintained below the safety threshold of 1,000 mL. In 9 patients, a balloon catheter was placed in the uterine cavity postoperatively to control bleeding and was removed within 24 h after surgery.

### 2.3 Follow-up and observation criteria

Complete resection (R0) is defined as the absence of residual lesions on imaging (ultrasound/MRI) 1 month postoperatively (10, 11).

Patients were followed up at 1, 6, and 12 months postoperatively, and annually thereafter. The follow-up period ends in June 2025. The symptom relief rate was assessed by comparing preoperative and postoperative hemoglobin levels, menstrual blood loss, and changes in pelvic pressure symptoms. Assessments included (12, 13): (1) gynecological examination; (2) pelvic ultrasound; (3) symptom questionnaire: Menstrual blood loss was quantified using the Pictorial Bleeding Assessment Chart (PBAC) (menorrhagia defined as PBAC ≥ 100), and pelvic pain/pressure symptoms were assessed using a Visual Analog Scale (VAS, 0–10 points); (4) Laboratory tests: Complete blood count to monitor hemoglobin levels; (5) Recurrence assessment: Recurrence is defined as the detection of a new myoma on imaging studies (ultrasound/MRI) that is pathologically confirmed as ALM following reoperation.

### 2.4 Statistical methods

Statistical analysis was performed using GraphPad Prism 10 software. Quantitative data are expressed as the median (range) or mean ± standard deviation (SD), and categorical data are expressed as n (%). For normally distributed continuous data (preoperative vs. postoperative hemoglobin levels), a paired *t*-test was used, with *p* < 0.05 considered statistically significant. For skewed data such as intraoperative blood loss, only descriptive statistics (median, range) are reported without inferential comparisons, given the small sample sizes and non-randomized assignment of surgical approaches. Categorical data are presented as n (%).

## 3 Results

### 3.1 General characteristics

The 20 patients ranged in age from 27 to 66 years, with a mean age of 42.5 ± 9.6 years. 18 cases (90%) were premenopausal, while 2 cases (10%) presented with new-onset masses postmenopausally. The primary clinical presentations were abnormal uterine bleeding with anemia (55%) and pelvic pain or pressure symptoms (30%); 3 cases (15%) were asymptomatic and were detected during routine physical examinations. Tumor markers CA125, CEA, CA19-9, and AFP were tested in all patients. Results were within the normal range in 17 cases (85%). Three cases showed mildly elevated CA125 levels (174.5, 114.4, and 50.2 U/mL), each of which was complicated by adenomyosis. This finding is consistent with previous reports that CA125 may be elevated in cases of ALM complicated by adenomyosis (14). Other baseline characteristics are summarized in Table 1.

**TABLE 1:** General clinical data of 20 patients with uterine ALM.

Characteristic	Value or number of cases (%)
Age (years, mean ± standard deviation)	42.5 ± 9.6
Parity	16 (80.0%)
No history of childbirth	4 (20.0%)
History of cesarean section	7 (35.0%)
Postmenopausal	2 (10.0%)
Clinical presentation	
Abnormal uterine bleeding with anemia	11 (55.0%)
Symptoms of pelvic compression	6 (30.0%)
Asymptomatic	3 (15.0%)
Gynecological examination	
Palpable pelvic mass (diameter 5.0–20.0 cm)	5 (25.0%)
Uterus enlarged to the size of a 6–12-week pregnancy	6 (30.0%)
Uterine myoma prolapsing into the vagina (diameter 6.0 cm)	2 (10.0%)
No obvious positive findings	7 (35.0%)
Complications	
Adenomyosis	6 (30.0%)
Ovarian cysts	4 (20.0%)
Endometrial polyps	2 (10.0%)

### 3.2 Imaging findings

**FIGURE 1:**
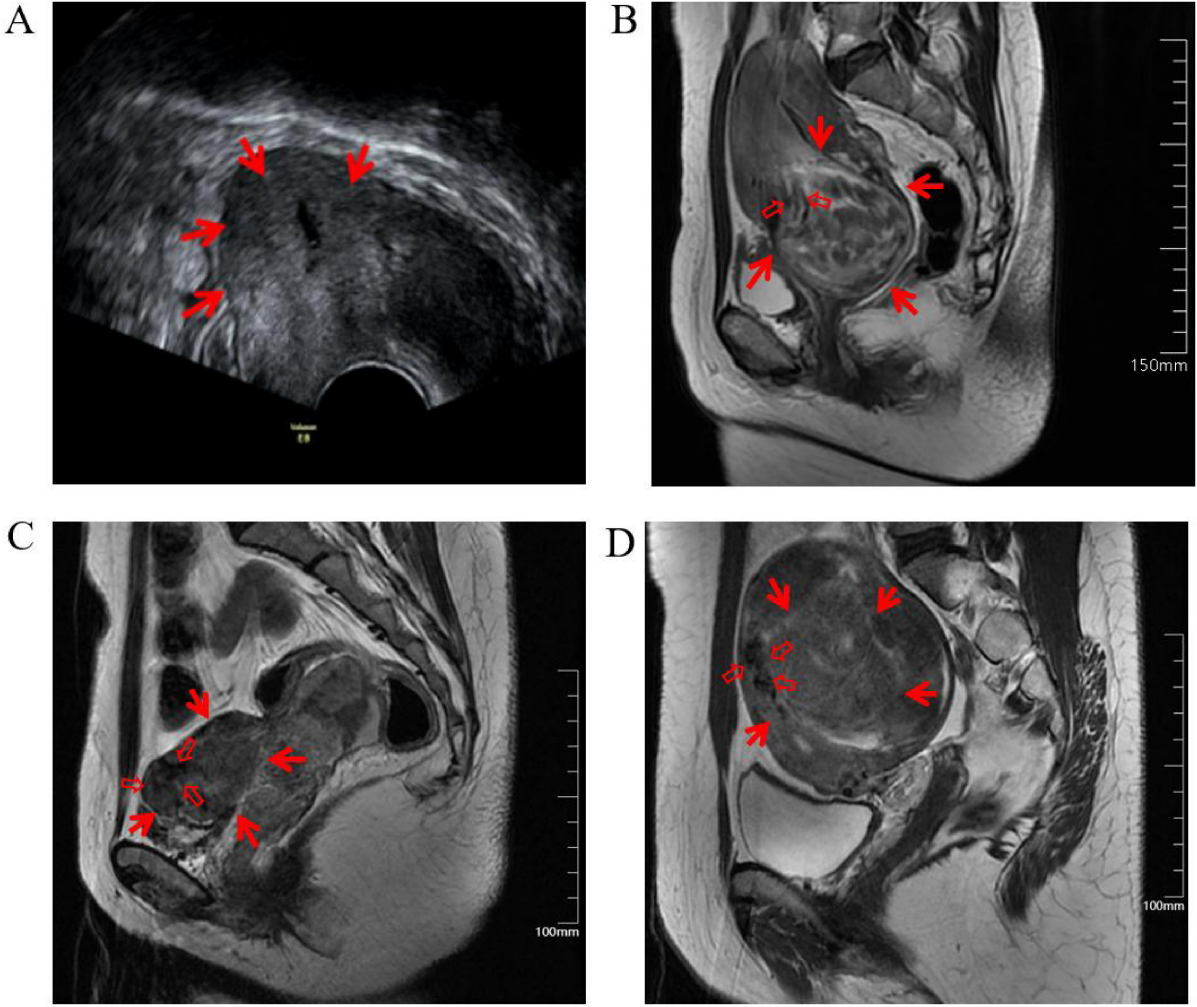
Preoperative imaging findings of ALM. **(A)** Preoperative ultrasound image: A space-occupying lesion measuring approximately 5.1 cm × 4.1 cm × 2.5 cm is seen in the right uterine wall, with tortuous and dilated peritumoral veins surrounding the lesion in an “worm-like” pattern. **(B)** Type 0 submucosal angioleiomyoma on pelvic MRI T2WI: A mass measuring approximately 6.0 cm × 5.8 cm × 5.0 cm is observed in the submucosa of the anterior uterine wall, partially protruding into the vagina, with abundant cord-like vascular signals within the tumor; the solid arrow indicates the main tumor body, and the open arrow indicates abnormal vessels. **(C)** Broad ligament angioleiomyoma on pelvic MRI T2WI: Multiple fused nodular lesions measuring approximately 9.2 cm × 4.7 cm × 4.2 cm are seen adjacent to the left uterine horn, accompanied by local necrosis, with coarse vascular signals visible at the lesion margin; the solid arrow indicates the main tumor body, and the open arrow indicates abnormal vessels. **(D)** Intramural angioleiomyoma on pelvic MRI T2WI: A round-like nodule measuring approximately 7.8 cm × 7.9 cm × 6.4 cm is seen in the posterior wall of the uterine fundus, with a cluster of coarse and tortuous vessels at the margin; the solid arrow indicates the main tumor body, and the open arrow indicates abnormal vessels.

Imaging evaluation primarily consisted of ultrasound and MRI. Ultrasound findings: 10 lesions were located in the uterine cavity or cervical canal, 5 were intramural or subserosal, and 5 were in the broad ligament. The maximum diameter ranged from 0.6 to 20.0 cm, with a median of 10.2 cm, including 5 cases of giant masses (diameter ≥ 10 cm). Multiple nodules were found in 9 cases, and solitary nodules in 11 cases. Endometrial thickening was noted in 2 cases. Heterogeneous hypoechoic lesions were observed in 15 cases (75%), homogeneous hypoechoic lesions in 2 cases (10%), and heterogeneous echoes with degenerative changes in 3 cases (15%). The resistance index (RI) was 0.65 ± 0.12 in 20 patients, with 4 cases (20%) having an RI < 0.5 (indicating a highly vascularized lesion), which is an important radiological clue for ALM (15). In this series, only one case was preoperatively suspected as ALM on ultrasound; the lesion was located on the right posterior wall and protruded outward, appearing cord-like along the course of the vessels, measuring 5.1 × 4.1 × 2.5 cm (Figure 1A). All other cases were definitively diagnosed by surgical pathology. The preoperative diagnosis rate was only 5%, which is consistent with the preoperative diagnosis rates of < 10% reported in domestic and international studies (16).

MRI features: 15 cases (75%) demonstrated significant progressive enhancement, while 5 cases (25%) showed heterogeneous enhancement with cystic changes. T2-weighted imaging (T2WI) revealed mixed signal intensity in 4 cases, and diffusion-weighted imaging (DWI) showed high signal intensity in 3 cases. Typical MRI images are shown in Figures 1B–D.

### 3.3 Surgical characteristics

The symptoms and treatment of uterine angioleiomyoma vary depending on the tumor location. Therefore, we analyzed clinical manifestations and treatment approaches by location. Surgical procedures, blood loss, and complications are summarized in Table 2. During hysteroscopy, the stalk of the lesion was located in the endocervical canal in one case, and the remaining nine cases were located in the uterine cavity. Four cases were FIGO type 0 submucosal myomas, of which two lesions prolapsed from the cervical canal into the vagina (both with a maximum diameter of 6 cm), and the other two measured 2.8 cm and 2.9 cm in diameter, respectively. There were three cases each of type 1 and type 2 submucosal myomas, with diameters ranging from 2.4 to 3.4 cm. Postoperative hemostasis was achieved using an intrauterine balloon in nine cases, with no instances of delayed bleeding. In all patients in this study, the fluid deficit was maintained below 1,000 mL. The median intraoperative blood loss was 15 mL (range 5–50 mL), and the mean operative time was 44.2 ± 29.8 min. One patient (Case 3) required two hysteroscopic procedures to resect the lesion due to a strong desire to preserve fertility and unclear lesion margins (details are provided in section 3.6); the remaining nine patients underwent complete macroscopic resection in a single procedure. No severe complications such as uterine perforation or transurethral resection of the prostate (TURP) syndrome occurred in the hysteroscopic group.

**TABLE 2:** Characteristics of 20 uterine ALM surgeries.

Myoma location (FIGO type)	Number of cases	Surgical approach	Tumor diameter (cm)	Intraoperative blood loss [mL, median (Q25, Q75)]	Operating time (mean ± SD)	Pathological subtype
Intrauterine (type 0–2)	10	Hysteroscopic surgery	≤6 cm	15 (5,50)	44.2 ± 29.8	Common type (9), cellular type (1), degenerative type(0)
Intramural/ subserosal (type 3–8)	5	Laparoscopic surgery (2 myomectomies + 3 total hysterectomies)	<10 cm	17 (10, 60)	84.6 ± 38.7	Common type (2), cellular type (1), degenerative type (2)
Other complex types	5	Open surgery (2 myomectomies + 3 total hysterectomies)	≥10 cm	300 (50, 1500)	135.8 ± 31.1	Common type (3), cellular type (1), degenerative type (1)

In the open surgery group, the median blood loss was 300 mL (range 50–1,500 mL). Two cases had intraoperative blood loss exceeding 1,000 mL and required intraoperative blood transfusion. However, because open surgery was preferentially selected for patients with larger myomas (diameter ≥ 10 cm), broad ligament location, or suspected malignancy, the higher blood loss in this group likely reflects greater case complexity rather than an inherent disadvantage of the open surgical approach (17). No direct statistical comparison was performed between groups due to non-randomized assignment and small sample sizes. One case developed postoperative fat liquefaction at the incision site, which healed after dressing changes. No intraoperative complications occurred in the laparoscopic surgery group.

### 3.4 Pathological findings

**FIGURE 2:**
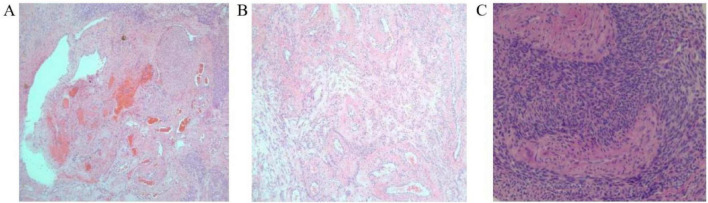
Histomorphology of uterine angioleiomyoma (HE staining). **(A)** Usual type. **(B)** Degenerative type. **(C)** Cellular type.

All cases exhibited the characteristic morphology of angioleiomyoma. Tumor sections appeared grayish-white to grayish-red, with a firm consistency and a distinct vascular network. All cases were diagnosed as ALM. Typical histological features are shown in Figures 2A–C.

Immunohistochemical results showed that smooth muscle markers (SMA and caldesmon) were diffusely positive in all 20 cases, while desmin was positive in 19 of 20 cases. The melanocytic marker HMB45 was negative in all 20 cases. No epithelioid cell morphology was observed on hematoxylin and eosin (HE) staining. Estrogen receptor (ER) and progesterone receptor (PR) testing results are detailed in Table 3. Adenomyosis was present concurrently in 6 cases (30%).

**TABLE 3:** Immunohistochemical characteristics of 20 cases of uterine ALM.

Pathological type (number of cases)	SMA	Desmin	ER/PR	Ki-67 Index	HMB45
Common type (*n* = 14)	14+	13+	13+	<5%	14−
Cellular type (*n* = 3)	3+	3+	2+	Focal 5–10%	3−
Degenerative type (*n* = 3)	3+	3+	2+	<5%	3−

### 3.5 Efficacy and follow-up results

In this study, 19 patients completed follow-up, with a median follow-up duration of 33.5 months (range: 22–67 months). Symptom relief, complete resection rates, recurrence rates, and pregnancy outcomes are summarized in Table 4.

**TABLE 4:** Summary of efficacy and follow-up results for 19 ALM patients.

Evaluation criteria	Preoperative	Postoperative (6 months/last follow-up)	Results
Anemia (*n* = 11)	Hb 90.6 ± 7.9 g/L	Hb 122.5 ± 10.3 g/L	*P* < 0.01
Anemia resolution rate	–	11/11 (100%)	All patients achieved hemoglobin levels ≥ 110 g/L
Menorrhagia (PBAC ≥ 100, *n* = 11)	PBAC ≥ 100	PBAC < 75 (all)	Return to normal menstrual flow
Pelvic pain/pressure (VAS, *n* = 6)	Moderate to severe	5 cases of complete remission (VAS = 0); 1 case of mild remission (VAS = 2)	Overall response rate: 100%
Compression symptoms (*n* = 6)	Dysuria (3), bloating/constipation (3)	5 cases of complete remission (83.3%); 1 case of marked improvement (16.7%)	–
Complete resection (R0)	–	19/20 (95.0%)	One case in the plan underwent partial resection due to fertility preservation needs (Case 3)
Recurrence	–	1/19 (5.3%)	Cellular type, detected at 6 years

Complete resection and recurrence: R0 resection was achieved in 19 of 20 patients (95%). One patient (Case 3) underwent planned incomplete resection to preserve fertility. Among the 19 patients who completed follow-up, one patient (after 6 years) experienced recurrence (5.3%). This patient had the ALM cellular type (Case 2) and was pathologically confirmed to have a new submucosal ALM. However, it‘s important to note that the definition of recurrence used in this study (pathologically confirmed after reoperation) may underestimate the true recurrence rate, as cases with imaging-only recurrence or residual regrowth without reoperation were not captured.

Symptomatic relief: Among the 11 patients with preoperative anemia (hemoglobin < 110 g/L), the mean hemoglobin level increased significantly at 6 months postoperatively, rising from 90.6 ± 7.9 g/L to 122.5 ± 10.3 g/L (paired *t*-test, *P* < 0.01), with all patients returning to normal hemoglobin levels ( ≥ 110 g/L). All 11 patients who had symptoms of menorrhagia [Pictorial Bleeding Assessment Chart (PBAC) score ≥ 100] preoperatively had PBAC scores below 75 at 6 months postoperatively. Among the 6 patients with moderate to severe pelvic pain or pressure (preoperative VAS score of 4–8), 5 patients experienced complete resolution of pain (VAS score = 0) at 6 months postoperatively, and 1 patient’s pain was reduced to mild (VAS score = 2). Pressure symptoms completely disappeared in 5 patients (two of whom had difficulty urinating and three of whom had abdominal distension/constipation), and symptoms improved significantly in one patient.

Pregnancy outcomes: Two patients achieved spontaneous conception after surgery. Case 3 (cellular type who underwent staged resection) conceived spontaneously 9 months postoperatively and delivered successfully via cesarean section. Another patient (common type) also achieved spontaneous conception during follow-up and showed no signs of recurrence.

### 3.6 Special cases

**FIGURE 3:**
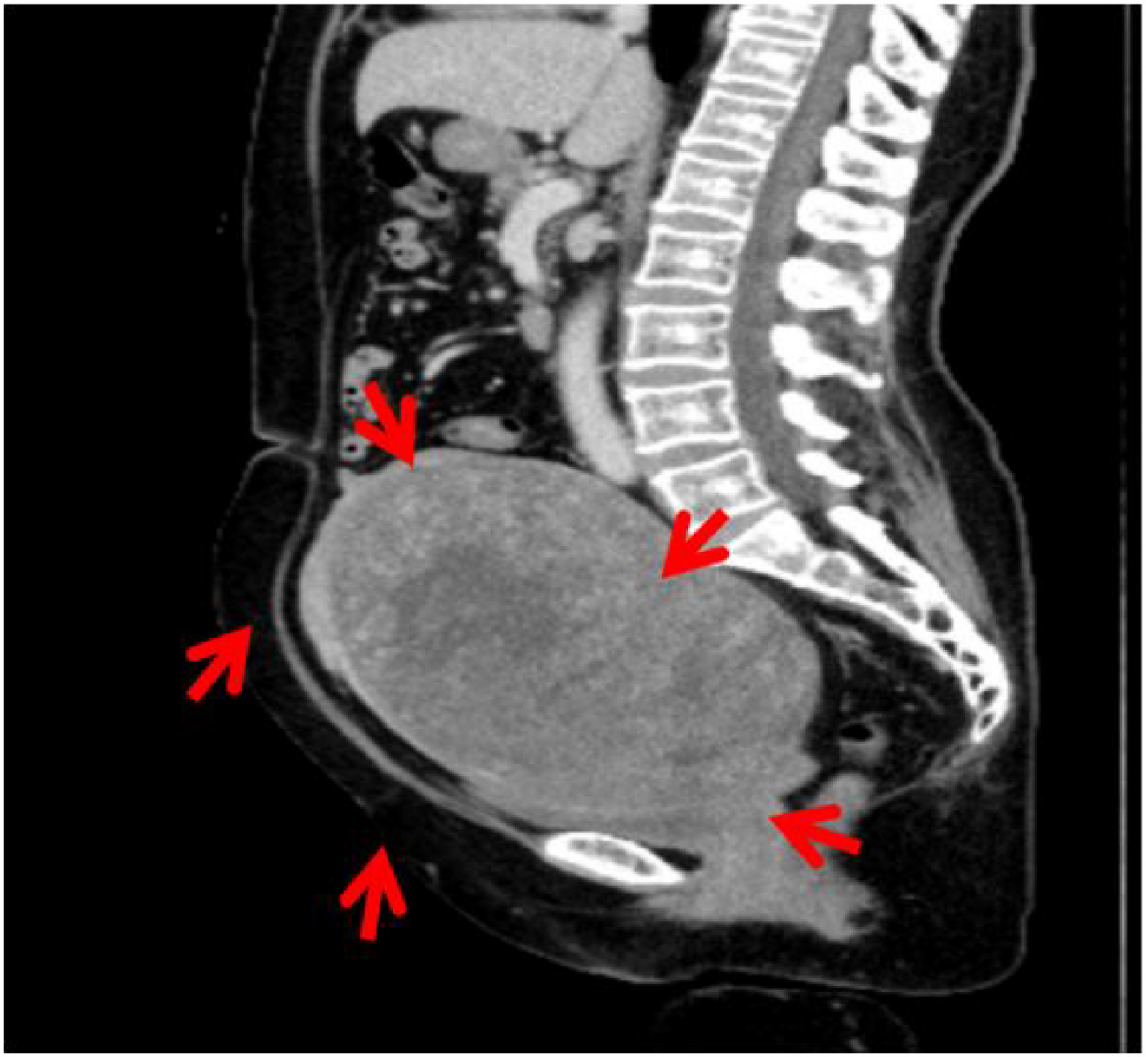
Enhanced abdominopelvic CT of Case 1: A round-like soft-tissue mass measuring 17.8 cm × 15.1 cm × 12.3 cm is seen in the abdominal cavity with heterogeneous density, showing multiple tortuous thin vascular signals and non-enhanced liquefactive necrotic areas.

Case 1 (Postmenopausal Giant ALM with Complications): A 66-year-old patient, 11 years postmenopausal, presented with swelling in both lower limbs. Ultrasound revealed a giant cystic-solid pelvic mass measuring 15.6 cm × 18.4 cm × 12.5 cm, with multiple cystic areas. CT suggested liquefactive necrosis (Figure 3), while MRI showed heterogeneous T2 signal intensity with areas of non-enhancement. Following anticoagulation and placement of an inferior vena cava filter, the patient underwent open total hysterectomy and bilateral salpingo-oophorectomy (blood loss: 700 mL). Pathology confirmed ALM with cystic components (ER/PR + ).

Case 2 (Recurrence of cellular type ALM): A 30-year-old female presented with menorrhagia. Ultrasound revealed an intrauterine hypoechoic nodule measuring 3.2 cm × 2.0 cm × 2.1 cm. She underwent hysteroscopic submucosal myomectomy. Pathology reported cellular ALM (focally P16-positive, Ki-67 3% positive, ER 2 + ). Four years postoperatively, follow-up ultrasound revealed an intrauterine hypoechoic nodule measuring 1.2 cm × 2.1 cm × 1.8 cm. Six years postoperatively, the lesion had enlarged to 3.7 cm × 3.0 cm × 2.5 cm, and a second hysteroscopic submucosal myomectomy was performed. Pathology again revealed an ALM (4 mitoses per 10 high-power fields), meeting the definition of tumor recurrence.

Case 3 (Pregnancy): A 29-year-old patient presented with menorrhagia and anemia. Preoperative ultrasound revealed a moderately echogenic intrauterine mass measuring 2.4 cm × 2.7 cm × 1.6 cm. MRI showed an irregular intrauterine mass measuring 4.7 cm × 2.0 cm × 1.8 cm (long T1 isointense, T2 mixed signal, well-defined margins, progressive enhancement). During the initial hysteroscopy, a soft nodule measuring approximately 2.5 cm × 2.0 cm was observed on the left uterine wall. Upon incision, the lesion was found to be a cystic cavity lined by a reticular vascular network and stroma, penetrating the myometrium with indistinct myometrial borders. As the patient desired future childbearing, ultrasound-guided debulking was performed in stages until the lesion’s surface was flush with the uterine wall, leaving approximately one-third of the lesion in situ. Postoperative pathology: (Uterine) cellular submucosal ALM, Ki-67 (3%+), ER (2+). The patient conceived naturally 9 months postoperatively. During pregnancy, ultrasound monitoring showed the myomatous nodule on the left uterine wall gradually enlarging from 0.9 to 3.8 cm. A cesarean section was performed at 39 weeks of gestation; no myoma was palpated intraoperatively. An ultrasound 42 days postpartum revealed a 2.4 cm myoma nodule in the left uterine wall. The patient breastfed for 6 months. At the 1-year postpartum follow-up, the fibroid had shrunk to 2.3 cm × 2.6 cm × 1.9 cm. MRI showed a round submucosal mass in the uterine body with isointense signal on T1 and hypointense signal on T2, measuring 3.0 cm × 3.0 cm × 1.7 cm. A second surgery completely resected the residual tissue, and the histopathology revealed a transformation to a typical leiomyoma (CD10-). These findings raise the hypothesis that pregnancy may influence the biological behavior of leiomyomas, but this observation is based on a single case and requires further validation.

## 4 Discussion

The clinical significance of uterine ALM lies in the discrepancy between its benign histology and potentially aggressive clinical behavior. Although ALM is histologically benign, its inherent rich vascularity complicates the diagnostic and therapeutic continuum, leading to three major clinical challenges: (1) low preoperative detection rates and frequent misdiagnosis, (2) a significantly increased risk of intraoperative bleeding, and (3) recurrence in certain pathological subtypes. The lack of large-scale clinical studies means that optimal management strategies for these issues remain unclear. Although this study is a retrospective analysis, the cohort provides practical, data-driven evidence that can help address these challenges.

### 4.1 Dual diagnostic and therapeutic challenges driven by the rich blood supply of ALM

Data from this cohort indicate that the clinical symptoms of uterine ALM (abnormal uterine bleeding in 55% of cases and pelvic pressure symptoms in 30% of cases) are similar to those of typical uterine myoma. This similarity largely explains the extremely low preoperative diagnosis rate, which was only 5% in this study. As a defining pathological feature, rich vascularity not only leads to diagnostic ambiguity but also increases perioperative risks (1). In this cohort, as many as 95% (19/20) of cases were misdiagnosed preoperatively. When imaging studies, particularly Doppler ultrasound, reveal a rich blood supply (RI < 0.5), this often creates a clinical dilemma. If this is mistakenly interpreted as a typical uterine fibroid, the surgical risk may be underestimated. Conversely, if rich vascularity leads to unwarranted suspicion of malignant tumors such as sarcomas, patients may undergo unnecessary testing and treatment. In our cohort, we encountered one case in which ALM was suspected preoperatively due to the presence of characteristic tortuous vessels (Figure 1). This case underscores the importance of recognizing this specific imaging pattern to prevent misdiagnosis. Rich vascularity poses a significant risk of uncontrollable bleeding during surgery. In our study, the median blood loss in the open surgery group was 300 mL, significantly higher than the blood loss observed in conventional myomectomies. Notably, 40% of these cases experienced blood loss exceeding 1,000 mL. Minimally invasive surgery also carries a risk of bleeding, requiring the surgeon to possess advanced hemostatic skills and an effective contingency plan. Therefore, the primary impact of uterine ALM on patients lies in the decision-making dilemma caused by diagnostic uncertainty, as well as the perioperative safety risks associated with its rich vascular supply.

### 4.2 Imaging evaluation aids in preoperative diagnosis

To improve preoperative detection rates, we compared our findings with the known imaging characteristics of conventional leiomyomas reported in the literature (1, 15, 16). On ultrasound, conventional leiomyomas typically appear as well-defined, homogeneous hypoechoic masses with a characteristic whorl-like or spiral structure. Color Doppler ultrasound typically reveals a peripheral or marginal vascular distribution (“basket” or “ring” sign), and the resistance index (RI) is almost always greater than 0.5, reflecting their composition primarily of smooth muscle and relatively sparse vascular distribution. In contrast, our ALM cases exhibited several distinctive features: (1) heterogeneous echogenicity, with degenerative changes present in 15% of cases—a finding uncommon in non-degenerative conventional leiomyomas; (2) A resistance index (RI) below 0.5 in 20% of ALMs—a finding that is extremely rare in conventional leiomyomas and should raise suspicion of a highly vascular tumor; (3) In one case, twisted, dilated veins were present around the tumor, presenting a “worm-like” appearance, which is a highly specific but insensitive sign almost never seen in conventional leiomyomas.

On magnetic resonance imaging (MRI), typical leiomyomas usually have well-defined borders and appear as homogeneous low signal on T2-weighted imaging (T2WI), often with a swirl-like appearance. On contrast-enhanced images, they exhibit mild to moderate homogeneous enhancement, with intensity similar to or slightly lower than that of the uterine myometrium. In our ALM cohort, 20% of cases showed mixed or heterogeneous T2WI signal—a finding attributed to the intermingling of smooth muscle bundles and irregular vascular channels. More importantly, 75% of our ALM cases demonstrated marked progressive enhancement (intense, sustained contrast uptake), which differs significantly from the mild enhancement pattern seen in conventional leiomyomas. In one case, large tortuous peritumoral feeding vessels were observed, most clearly visualized on contrast-enhanced MRI, a unique feature in the presence of ALM. However, degenerating conventional leiomyomas (such as liquefied, cystic, or red degeneration types) may occasionally exhibit heterogeneous T2WI signal and varying degrees of enhancement, leading to confusion with ALM. Therefore, these radiological features should be interpreted in conjunction with the clinical context, and pathological confirmation remains essential.

Furthermore, in our specific study population, the proportion of complex cases was relatively high. Our study population included: multiple myoma (9 cases, 45%), giant myoma (≥10 cm, 5 cases, 25%), broad ligament myoma (5 cases, 25%), patients with concurrent endometrial thickening (2 cases), and patients with postmenopausal new-onset myoma (2 cases). In this specific context, we believe that preoperative MRI has significant clinical value and is cost-effective. First, magnetic resonance imaging (MRI) allows us to prospectively identify features specific to ALM—particularly the “marked progressive enhancement” pattern (present in 75% of the ALM cases in our study, compared to only 30% in conventional uterine myoma), which substantially increases the preoperative suspicion of a highly perfused tumor. Second, MRI accurately delineates lesion margins and the extent of myometrial involvement, which is crucial for surgical planning, particularly in fertility-sparing cases (e.g., Case 3). Third, MRI helps rule out sarcomas by revealing benign features (well-defined margins, progressive rather than rapid heterogeneous enhancement), thereby avoiding unnecessary radical surgery. However, we emphasize that this recommendation applies only to complex cases and cannot be generalized to all patients with simple, asymptomatic leiomyomas, in whom ultrasound alone is sufficient.

### 4.3 Targeted diagnostic workflow and stratified surgical strategy

To address diagnostic challenges, our experience supports a stepwise workflow: transvaginal ultrasound (TVS) screening → MRI evaluation → intraoperative assessment → pathological confirmation. The core contribution lies not in the novelty of the workflow, but in the unique emphasis placed on each step. For ALM, initial TVS screening should go beyond measuring size and location to actively identify signs of rich vascularity (low RI values) and characteristic vascular morphology, such as tortuous “worm-like” vessels (15). MRI can further distinguish the gradual enhancement pattern of ALM from the rapid, heterogeneous enhancement commonly seen in sarcomas, and can more accurately assess lesion margins and relationships with adjacent structures to support surgical planning. Strengthening this “risk-stratification” approach can significantly improve the preoperative suspected diagnosis rate.

Regarding surgical strategies, we established clear selection criteria (based on FIGO classification, tumor size and location, and the patient’s desire for future childbearing) and validated the safety of this approach. Data indicated that hysteroscopic surgery is safe and feasible for submucosal myoma of types 0–2 with a diameter ≤ 6 cm. For intramural or subserosal myoma (types 3–7) with a diameter < 10 cm, laparoscopic surgery is safe and feasible. For uterine myoma ≥ 10 cm in diameter, broad ligament myoma (type 8), lesions with preoperative suspicion of malignancy, or multiple large myoma, open surgery provides better exposure and improved hemostasis (17). In this cohort, both cases of massive hemorrhage occurred during open surgery for giant ALMs. This underscores the necessity of thorough preoperative assessment of tumor size and blood supply, as well as the active selection of an open surgical approach for giant or highly vascularized ALMs. For high-risk cases, plans for blood transfusion and vascular ligation should be established in advance (17).

Our experience with hysteroscopic resection of submucosal ALMs measuring up to 6 cm in diameter indicates that our surgical strategy aligns with the fundamental principles of current clinical guidelines, while also expanding the boundaries of safe resection under specific conditions. According to previous guidelines (such as the 2024 ESGE guidelines), a staged surgical approach is typically recommended for larger FIGO type 1–2 submucosal myomas (diameter > 3–5 cm) to reduce the risk of fluid overload and uterine perforation (18, 19). In our series of 10 consecutive procedures, all myoma with diameters ranging from 3 to 6 cm were completely resected in a single step. The mean blood loss was only 15 mL, and intraoperative fluid deficit was consistently maintained below the safety threshold of 1,000 mL. No complications, such as uterine perforation or transurethral resection of the prostate (TURP) syndrome, occurred. All 10 hysteroscopic procedures in this study were performed by an experienced senior surgeon. Real-time ultrasound guidance was maintained throughout the procedure (20), with strict monitoring of fluid balance and patient vital signs. Postoperative intrauterine balloon compression was used for hemostasis, ensuring surgical safety to the greatest extent possible. Our cohort’s experience indicates that individualized treatment strategies should be employed for the hysteroscopic resection of submucosal myoma, emphasizing the importance of surgeon experience and patient selection.

It is important to emphasize that our data do not support the superiority of any single surgical approach over others. The choice of surgical approach should be individualized based on tumor characteristics and patient factors, and the observed differences in intraoperative blood loss primarily reflect differences in case complexity rather than the inherent safety or efficacy of the surgical technique itself.

### 4.4 Pathological subtypes, R0 resection, and recurrence risk: focus on cellular type

Pathological analysis in this study revealed the heterogeneity of ALM. The majority (17/20) were of the typical type or accompanied by degenerative changes, exhibiting low proliferative activity (Ki-67 < 5%) and a favorable prognosis. Notably, the cellular subtype (3/20, 15%) warrants particular attention. The sole patient who experienced recurrence belonged to this subtype, and the Ki-67 index showed focal elevation (5–10%), suggesting that the cellular subtype may be associated with a higher risk of recurrence (21). This observation suggests two points that should be considered preliminary and hypothesis-generating. First, achieving R0 resection may be particularly important for potentially reducing the risk of recurrence, especially for cellular-type ALM, and postoperative imaging to confirm the absence of residual lesions appears prudent (22). Second, although firm evidence is lacking due to the small number of recurrent cases, stratifying follow-up according to pathological subtype could be considered based on our limited experience. Patients with cellular-type ALM may warrant closer monitoring and longer follow-up periods. However, given that this finding is based on only one recurrent case among three cellular-type ALMs, it should be interpreted as hypothesis-generating and requires validation in larger, prospective cohorts.

### 4.5 Considerations regarding fertility preservation and outcomes

Two young patients in this cohort achieved spontaneous pregnancy postoperatively, with one delivering at term, confirming the feasibility of fertility-preserving surgery for ALM. The clinical course of Case 3 is noteworthy. To preserve fertility, partial resection of the lesion was performed during the initial hysteroscopic surgery. The patient subsequently conceived spontaneously and delivered at term. During pregnancy, the residual lesion enlarged, and postpartum menstruation resumed with heavy bleeding. A second hysteroscopic procedure was performed to remove the residual tissue; unexpectedly, histopathological examination revealed a typical leiomyoma. Although this rare finding does not allow for definitive conclusions, it raises the possibility that the hormonal microenvironment during pregnancy may influence the biological behavior of ALM and provides valuable clinical clues for future research on hormonal regulation of ALM. It is hypothesized that elevated levels of progesterone during pregnancy may modulate the ER/PR signaling pathway, thereby inhibiting abnormal angiogenesis and cell proliferation in ALMs and promoting their transformation into ordinary leiomyomas (23, 24). Future studies with larger sample sizes and mechanistic investigations (e.g., hormonal receptor profiling, transcriptomic analysis) are needed to explore whether pregnancy or hormonal interventions truly influence ALM biology. At the same time, it reminds us that for ALM patients with a desire for childbearing, treatment decisions should strike an optimal balance between lesion resection and fertility preservation, and it is necessary to strengthen monitoring of uterine myoma during pregnancy and the postpartum period.

### 4.6 Exploration of potential biomarkers for differential diagnosis

Distinguishing ALM from uterine perivascular epithelioid cell tumor (PEComa) is clinically important due to their different biological behaviors. Although PEComa may occasionally exhibit vascular structures, it typically expresses melanocytic markers (HMB45, Melan-A, MiTF) with variable smooth muscle actin positivity, whereas ALM consistently demonstrates diffuse smooth muscle differentiation (25, 26). In this cohort, all 20 cases showed strong, diffuse expression of SMA and caldesmon with uniform HMB45 negativity, and no epithelioid cell morphology was observed—findings that strongly support the diagnosis of ALM. However, rare cases of HMB45-negative PEComa have been reported in the literature (27), and this retrospective study did not test for other melanocytic markers such as Melan-A and MiTF. Therefore, while our immunophenotype strongly favors ALM, HMB45-negative PEComa cannot be absolutely excluded. We recommend that future prospective studies routinely include Melan-A and MiTF testing to achieve a more reliable differential diagnosis.

ALM should also be distinguished from: (1) STUMP (smooth muscle tumor of uncertain malignant potential), which lacks the characteristic prominent vascular network of ALM and shows atypical histologic features; (2) Leiomyosarcoma, which demonstrates frank cytologic atypia, coagulative necrosis, and elevated mitotic index ( > 10–15/10 HPF)—none of which were observed in our cases; (3) Highly vascular conventional leiomyoma, which may mimic ALM but lacks the irregular, thick-walled vascular channels characteristic of ALM and typically has a resistance index > 0.5 on Doppler ultrasound.

The cellular-type ALMs in this cohort showed high ER/PR positivity (2/3 cases), suggesting hormone sensitivity and a potential rationale for molecular imaging techniques such as estrogen receptor imaging (16). Additionally, MED12 mutations occur in approximately 70% of conventional uterine leiomyomas but are extremely rare in ALM (3). If MED12 status could be determined non-invasively (e.g., via liquid biopsy or radiomics), it might serve as an adjunctive tool to distinguish highly vascular ALM from highly vascular conventional leiomyoma. The cellular ALM subgroup (3/20) exhibited focally elevated Ki-67 and included the only recurrence in this cohort, suggesting possible molecular heterogeneity. Systematic investigation of proliferation markers (p16, p53), cell cycle regulators (Cyclin D1), and apoptotic pathways in larger cohorts may enable molecular-based risk stratification in the future.

### 4.7 Comparison with published literature

To contextualize our findings, we compared them with available published case series and reports (1–3, 8, 11, 12, 14, 16, 17) (Supplementary Table 1).

The mean age in our cohort (42.5 years) was consistent with previous reports (range 37–44 years) (1, 2, 8, 11, 12, 14). Abnormal uterine bleeding was the most common symptom across studies (55–100%) (1–3, 11), and preoperative diagnosis rates were uniformly low (0–5%) (2). Submucosal lesions were more frequent in our cohort (50%) than in the largest prior series (32.6%) (2), likely reflecting referral bias for hysteroscopic surgery. Intramural/subserosal (25%) and broad ligament (25%) proportions were comparable to those reported by He and Jiang (36.0 and 19.1%, respectively) (2). Pathological subtype distribution in our cohort (common 70%, cellular 15%, degenerative 15%) was similar to that reported by He and Jiang (68 and 18%) (2) and Gupta et al. (11). All reported cases showed consistent immunophenotypes: diffuse positivity for smooth muscle markers (SMA, desmin, caldesmon) and negativity for HMB45 and Melan-A (1–3, 11, 12, 14, 17). The transfusion rate in our cohort (10%) was comparable to that reported by He and Jiang (6.7%) (2). Unusual presentations documented in the literature include a giant ALM with DIC (12), a 12.5 kg ALM with characteristic CT findings (8), pseudo-Meigs syndrome with CA-125 elevation (14), and a 40 cm ALM with septic metastases misdiagnosed as sarcoma on frozen section (17). Recurrence was rare across studies: 5.3% in our cohort (1/19, cellular type) and approximately 1–4% in He and Jiang (2). No recurrences were reported in other series (1, 3, 8, 11, 12, 14, 16, 17), though follow-up durations varied.

Other findings from this cohort, including those on surgical blood loss, recurrence, potential hormonal effects during pregnancy, and fertility outcomes, are discussed above and should be considered hypothesis-generating. Taken together, these findings provide descriptive data from a single-center experience that may inform future multicenter studies. Given the small sample size and retrospective design, the consistency across studies should not be overinterpreted as validation of clinical recommendations, but rather as reassurance that our cohort is broadly representative of previously reported uterine ALM cases.

This study has several limitations. First, the retrospective single-center design with a small sample size (*n* = 20) limits generalizability and statistical power. No control group was included. Second, surgical approach was not randomized, introducing selection bias. The higher blood loss observed in open surgery likely reflects greater case complexity (larger tumors, broad ligament location) rather than an inherent disadvantage of the surgical route itself. Third, median follow-up of 33.5 months may be insufficient to detect late recurrences. Recurrence was defined as pathologically confirmed reoperation, which likely underestimates the true recurrence rate by excluding imaging-only recurrences. Fourth, Melan-A and MiTF were not routinely tested; therefore, HMB45-negative PEComa cannot be completely excluded, although the immunophenotype strongly supports ALM. Fifth, referral bias exists as our institution is a tertiary center specializing in hysteroscopic and complex pelvic surgery, which may limit generalizability to community settings. Sixth, the observation of ALM transformation during pregnancy is based on a single case and is hypothesis-generating only. In conclusion, all findings should be interpreted as descriptive and hypothesis-generating, requiring validation in larger prospective multicenter studies.

### 4.8 Summary

Uterine ALM is a rare benign tumor whose defining feature—a rich vascular network—poses challenges for both preoperative diagnosis and intraoperative bleeding risk. Based on this single-center retrospective cohort of 20 cases, several observations emerge. First, preoperative diagnosis remains difficult; we propose a stepwise workflow (ultrasound screening → MRI evaluation → intraoperative assessment → pathological confirmation) with emphasis on identifying characteristic vascular imaging features (low RI on Doppler, tortuous peritumoral vessels, and marked progressive enhancement on MRI). Second, surgical planning should be individualized based on FIGO classification, tumor size and location, and fertility preferences. Third, while R0 resection appears important for reducing recurrence, this observation is based on limited data; the cellular subtype may be associated with higher recurrence risk, warranting closer follow-up. These findings are descriptive and hypothesis-generating, reflecting a single-center experience. Larger prospective multicenter studies with extended follow-up are needed to validate our observations and establish standardized management guidelines.

## Data Availability

The original contributions presented in the study are included in this article/Supplementary material, further inquiries can be directed to the corresponding author.
