# Supplementary material for: Clinical and pathological characteristics, diagnosis, and surgical treatment of uterine angioleiomyoma: a retrospective cohort study
Source: Front Med (Lausanne). 2026 Jun 4;13:1823201. doi: 10.3389/fmed.2026.1823201 (PMC13275716; doi:10.3389/fmed.2026.1823201)
Supplement: Supplementary file 1 [file Table_1.docx]

**TABLE S1 Summary of important literature on ALM**

| **Citation** | **Results** | **Key Features / Conclusions** |
| --- | --- | --- |
| **Pham T, et al.** *Int J Surg Pathol.* 2024;33(2):430-5. [1] | Age 44 years; Symptoms: AUB, anemia, pelvic organ prolapse; Preoperative CT showed enhancing lesion; Tumor 1.7 cm (left posterolateral myometrium); Pathology: venous type; IHC: SMA+, desmin+, caldesmon+, CD34+ (vessels), HMB45-, MelanA-; Treatment: TLH+BSO; Follow-up: no recurrence | Single case report; Contrast-enhanced CT helps distinguish ALM from conventional leiomyoma; IHC differentiates from PEComa |
| **He SL, Jiang JF.** *BMC Womens Health.* 2024;24(1):479. [2] | 89 cases; Mean age 41.8 years; AUB 68%; Preoperative diagnosis rate 0%; Location: uterine corpus 68.5% (submucosal 32.6%, intramural/subserosal 36.0%), broad ligament 19.1%, cervix 11.2%, vagina 1.1%; Multiple lesions 22.5%; Transfusion 6 cases (6.7%); Blood loss >500 mL in 6 cases; 59 uterus-preserving, 30 hysterectomy; Recurrence 1 case (~1-4%); Ki-67 <10% | Largest cohort study (89 cases); First report of vaginal ALM; Highlights intraoperative bleeding risk for multiple/large tumors; Recurrence is rare |
| **Kapaganti VK, et al.** *Indian J Pathol Microbiol.* 2024;67(4):877-80. [3] | 2 cases (31 and 45 years); Symptoms: menorrhagia, lower abdominal pain, dysmenorrhea; Ultrasound showed endometrial polyps; Tumor size 3 cm; Hysteroscopic polypectomy; Pathology: venous type; IHC: SMA+, desmin+, CD34+; Follow-up: no recurrence | Two case reports; Rare presentation as endometrial polyps; Differential diagnosis: myopericytoma, angiomyofibroblastoma, endometrial stromal tumor, PEComa |
| **Pierro A, et al.** *Radiol Case Rep.* 2018;13(2):371-5. [8] | Age 37 years; Symptoms: abdominal distension, menorrhagia, dyspepsia; CA125 304 IU/mL; Hb 9 g/dL; Tumor 32×30×25 cm, weight 12.5 kg; CT features: multiple vascular branches, "sand-like" enhancement, pelvic varicocele, uterine artery hypertrophy; Treatment: total hysterectomy; Pathology: smooth muscle bundles with thick-walled vessels; Follow-up: no recurrence | Single giant case report (12.5 kg); Proposes CT diagnostic triad: vascular branches, sand-like enhancement, uterine artery hypertrophy with pelvic varicocele |
| **Gupta M, et al.** *Int J Surg Pathol.* 2018;26(1):18-23. [11] | 6 cases; Age 32-46 years (median 39); AUB 100%; Location: corpus 4 cases (2 submucosal, 2 intramural), cervix 2 cases; Tumor size 3-15 cm; Pathology: solid+venous type (4), cavernous type (2); IHC: MSA+, desmin+, h-caldesmon+, CD10-, HMB45-, MelanA-, WT1-; Treatment: polypectomy (2), myomectomy (3), hysterectomy (1); Follow-up: uneventful | 6-case series; Supports WHO inclusion of ALM in uterine mesenchymal tumor classification; IHC differentiates from EST and PEComa |
| **Sato H, et al.** *BMC Womens Health.* 2023;23(1):157. [12] | Age 44 years; Symptoms: abdominal mass, anemia (no genital bleeding); DIC (Fib 94 mg/dL, FDP 374.5 μg/mL, D-dimer 81 μg/mL); Hb 8.3 g/dL; Tumor 23×16 cm, weight 2900 g; Intraoperative blood loss 1195 mL; Pathology: numerous thick-walled vessels with intraluminal thrombi; IHC: SMA+, desmin+, caldesmon+, HMB45-, CD10 (focal+), Ki-67 2%; Genetics: CCND2 and AR amplification; Coagulopathy resolved post-hysterectomy | First report of ALM with DIC; Proposed mechanism: blood stasis in tumor vessels → thrombosis → ischemic injury → procoagulant release → DIC |
| **Thomas S, et al.** *Case Rep Pathol.* 2012;2012:519473. [14] | Age 47 years; Symptoms: ascites, pleural effusion, CA125 477.1 IU/mL; Tumor 18×15×8 cm; Pathology: smooth muscle bundles with thick-walled vessels, focal cellular atypia (no atypical mitoses, no necrosis); IHC: SMA+, desmin+, HMB45-, Ki-67 <1%; Ascites and CA125 normalized post-surgery; Follow-up 6 months: no recurrence | First report of ALM with atypia + pseudo-Meigs syndrome + markedly elevated CA125; Emphasizes differentiation from PEComa and leiomyosarcoma |
| **Seth A, Mathur A.** *J Midlife Health.* 2021;12(2):179-84. [16] | 2 cases (32 and 47 years); Symptoms: menorrhagia, lower abdominal pain; Presentation: cervical polyp / endocervical mass; Tumor size 6 cm, 3.2 cm; Pathology: venous type with lipomatous component (cervical lesion); IHC: SMA+, CD34+, ER weak+, PR-, CD10-, S100-, HMB45-; Treatment: hysteroscopic polypectomy, total hysterectomy | Two case reports; Venous type with focal adipocytic metaplasia (first cervical ALM with fat); Supports WHO inclusion of ALM as benign variant of uterine leiomyoma |
| **Sánchez-Iglesias JL, et al.** *Arch Gynecol Obstet.* 2019;300(4):841-7. [17] | Age 47 years; Symptoms: fever, ascites, neck abscess (S. agalactiae infection); Tumor 40×30×35 cm; Frozen section misdiagnosed as endometrial stromal sarcoma; Treatment: hysterectomy + BSO + omentectomy + lymphadenectomy; Pathology: confirmed ALM; IHC: SMA+, desmin+, h-caldesmin+, ER+ (80%), PR+ (80%), CD34+, CD31+, CD10-, HMB45-, Ki-67 <5%; Follow-up 3 years: no recurrence | Single case + systematic review; Largest reported ALM (40 cm) with infection and septic metastases; Highlights limitations of frozen section and importance of IHC |
